# Identification of myxobacteria-derived HIV inhibitors by a high-throughput two-step infectivity assay

**DOI:** 10.1186/1475-2859-12-85

**Published:** 2013-09-24

**Authors:** Javier P Martinez, Bettina Hinkelmann, Eric Fleta-Soriano, Heinrich Steinmetz, Rolf Jansen, Juana Diez, Ronald Frank, Florenz Sasse, Andreas Meyerhans

**Affiliations:** 1 Infection Biology Group, Department of Experimental and Health Sciences, Universitat Pompeu Fabra, Barcelona, Spain; 2 Department of Chemical Biology, Helmholtz Centre for Infection Research, Braunschweig, Germany; 3 Department of Microbial Drugs, Helmholtz Centre for Infection Research, Braunschweig, Germany; 4 Molecular Virology Group, Department of Experimental and Health Sciences, Universitat Pompeu Fabra, Barcelona, Spain; 5 Institució Catalana de Recerca i Estudis Avançats (ICREA), Barcelona, Spain

## Abstract

**Background:**

Drug-resistance and therapy failure due to drug-drug interactions are the main challenges in current treatment against Human Immunodeficiency Virus (HIV) infection. As such, there is a continuous need for the development of new and more potent anti-HIV drugs. Here we established a high-throughput screen based on the highly permissive TZM-bl cell line to identify novel HIV inhibitors. The assay allows discriminating compounds acting on early and/or late steps of the HIV replication cycle.

**Results:**

The platform was used to screen a unique library of secondary metabolites derived from myxobacteria. Several hits with good anti-HIV profiles were identified. Five of the initial hits were tested for their antiviral potency. Four myxobacterial compounds, sulfangolid C, soraphen F, epothilon D and spirangien B, showed EC_50_ values in the nM range with SI > 15. Interestingly, we found a high amount of overlapping hits compared with a previous screen for Hepatitis C Virus (HCV) using the same library.

**Conclusion:**

The unique structures and mode-of-actions of these natural compounds make myxobacteria an attractive source of chemicals for the development of broad-spectrum antivirals. Further biological and structural studies of our initial hits might help recognize smaller drug-like derivatives that in turn could be synthesized and further optimized.

## Introduction

Current Human Immunodeficiency Virus (HIV) treatment comprises a combination of three or more antiretroviral drugs, which often lead to drug-resistance and therapy failure due to drug-drug interactions and toxic effects, especially in patients with HIV-associated co-infections [1-5]. As such, there is a continuous need for the development of new and more potent anti-HIV drugs. Here we describe the establishment of a two-step high-throughput screening (HTS) platform to identify molecules against HIV infection. The assay is based on the highly permissive TZM-bl cell line [6]. These are modified HeLa cells expressing endogenous CD4, CXCR4 and CCR5 receptors, and an integrated Tat-dependent firefly luciferase gene. The TZM-bl cells in combination with HIV pseudoviruses have been extensively used in antibody neutralization tests with highly reproducible results [7] and in a previous siRNA screen [8]. Upon infection, the viral RNA genome is reversed transcribed into DNA and integrated into the host-cell as a provirus. Then, the proviral-produced Tat protein mediates the activation of the LTR-driven luciferase gene. Thus, the amount of luciferase signal is in direct relationship with the efficiency of infection and the antiviral activity of test compounds can be measured as a function of reductions in luciferase expression compared to un-treated or drug-solvent controls.

**Figure 1:**
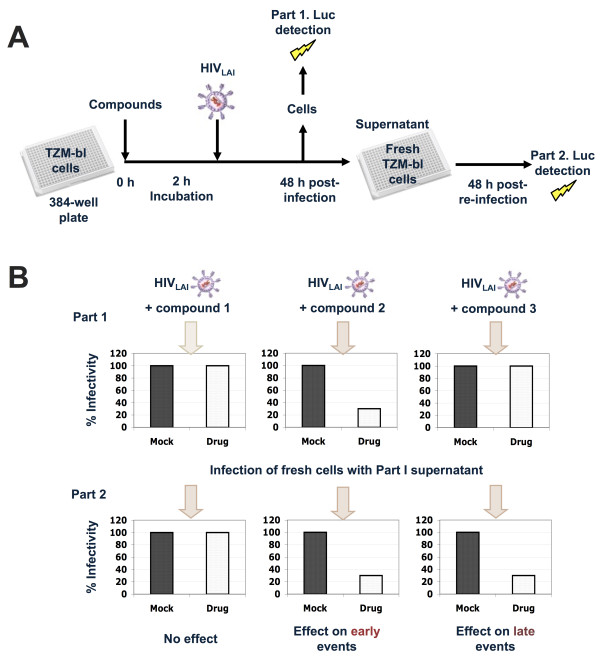
**Overview of the HIV screen assay. (A)** Two step infection approach used for the primary screen. In Part 1, TZM-bl cells are seeded on 384-well plates, incubated with the test compounds for 2 h and infected with HIV. 48 h post-infection, supernatants from infected cells are used to infect fresh TZM-bl cells (beginning of Part 2). Cells from Part 1 are assayed for Tat-dependent luciferase expression and, in parallel, for compound-related toxicity. 48 h after re-infection, cells of Part 2 are assayed in the same manner (See text for details). **(B)** Scheme showing the possible outcomes of the two-step HIV screen. A compound inactive against HIV will not show significant reductions in Tat-dependent luciferase expression compared to solvent controls for both Part 1 and Part 2 of the screen (left). A compound truly acting on early steps of the HIV cycle will show reductions in luciferase expression in Part 1 that will be reflected in Part 2 of the screen (middle). A compound acting on late events might show little or no decrease in relative luciferase in Part 1, but it will show a significant reduction of luciferase expression in cells assayed in Part 2.

The two-step cell-based screen is shown in Figure 1 and described in Materials and Methods. Briefly, TZM-bl cells seeded in 384-well plates are incubated with test compounds and infected with HIV_LAI_ at a multiplicity of infection (MOI) of 0.5. To monitor compound-related toxicity in parallel, TZM-bl cells are left uninfected and incubated with test compounds (Part 1 of the screen). 48 h after initial infection, virus-containing supernatants are used to infect fresh TZM-bl cells (Part 2) and cells from Part 1 are assayed for Tat-dependent luciferase expression (Figure 1A). Compound-related toxicity is quantified in parallel plates by a commercial ATP assay. 48 h after re-infection of fresh TZM-bl cells (part 2 of the screen), plates are assayed as in part 1. This approach is able to identify molecules acting on early HIV steps (from entry to translation) by detecting luciferase reductions in cells from Part 1, and compounds acting on late HIV steps (such as trafficking, assembly, release and maturation), which will be detected in re-infected cells of Part 2 of the screen (Figure 1B). The assay set-up is similar to a previous anti-HIV assay using MAGI cells [9]. The effects of test compounds on infectivity and cell viability are quantified by normalizing the mean luciferase expression units to the solvent controls and hits are determined by calculating a robust Z-score as described [9] (see Materials and Methods).

## Results and discussion

**Figure 2:**
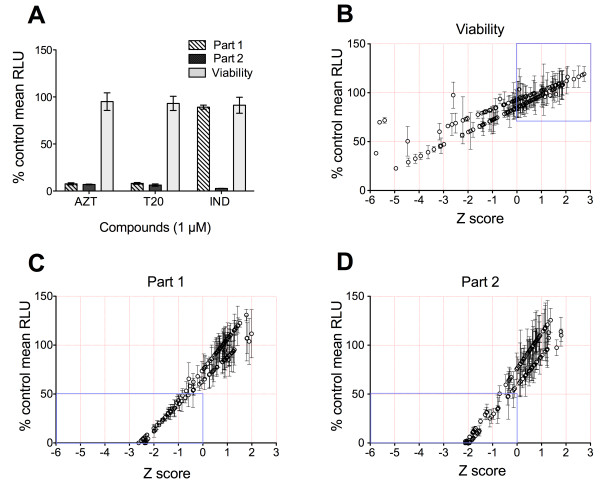
**Screen validation and hit identification. (A)** The screen protocol was validated with the known HIV inhibitors Zidovudine (AZT), Enfuvirtide (T20) and Indinavir (IND). Values are plotted as % mean luciferase expression relative to solvent controls. **(B-D)** The results of the myxobacterial library screen are shown for Viability **(B)**, infectivity Part 1 **(C)** and re-infection Part 2 **(D)**. For each test compound, the % mean RLU of the control is plotted vs. the calculated robust Z-score. Hits are defined as compounds inhibiting HIV by at least 50% of the untreated controls (infectivity Z-score < 0) and with less than 30% compound-related toxicity (viability Z-score > 0) (blue boxes). Error bars are standard deviations of quadruple measurements.

To validate the two-step screen, we tested the effect of three FDA-approved anti-HIV drugs: the nucleoside reverse transcriptase inhibitor Zidovudine, the fusion inhibitor Enfuvirtide and the protease inhibitor Indinavir on HIV infectivity and cell viability (Figure 2A). The assay yielded an excellent separation between solvent and drug controls (assay Z-factor of 0.9) [10]. The platform was then used to screen a small library of around 150 myxobacterial secondary metabolites from the Helmholtz Centre for Infection Research, Braunschweig, Germany [11,12]. Myxobacteria are among the top producers of natural products, matching those produced by marine bacteria [13]. These true microbial cell factories are known to manufacture highly active antimicrobials with novel chemical structures [14,15]. The library was screened using a single concentration of each test substance (and solvent controls) assayed in quadruple. Robust Z-score values were calculated from the mean of the replicates as described [9,16], and plotted against the% mean of the solvent controls. Hits are defined as compounds inhibiting 50% infectivity with Z-scores < 0 and, conversely, compounds having less than 70% toxic effects with Z-scores > 0 (Figure 2B-D).

**Figure 3:**
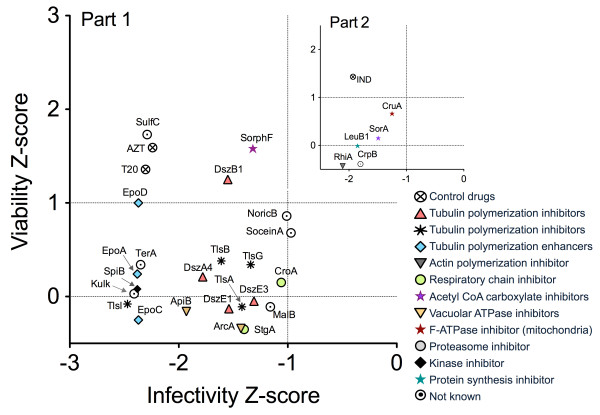
**Summary of the strongest myxobacterial hits.** The plot shows the viability Z-score vs. the infectivity Z-score of compounds having the strongest antiviral activity (Z score < -1) for both Part 1 and Part 2 (inlay). The figure shows a clear clustering of compounds based on structural similarities and known function (right). The commercial drugs AZT, T20 and IND are included as positive controls. Compounds are abbreviated to prevent figure congestion. Corresponding compound names are given in Table 1.

After discarding compounds without significant antiviral and/or toxic effects, we analyzed hit ranking by comparing their infectivity and viability Z-scores for both Part 1 and Part 2 of the screen (Figure 3). Among the top hits (i.e compounds having infectivity Z-scores < -1) in Part 1, we found clustering of derivatives of the tubulin polymerization inhibitors disorazoles, polyketides isolated from *Sorangium cellulosum*[17], and tubulysins, unusual peptides isolated from *Archangium gephyra*[18]. In the primary screen both compounds inhibited HIV by 80 - 90% with infectivity and viability Z-scores of < -1.3 and > 0, respectively (Table 1). The tubulin polymerization enhancers epothilones, macrolides isolated from *Sorangium cellulosum*[19], were also found among the strongest inhibitors in Part 1 of the screen (Figure 3 and Table 1). Other hits were the ATPase inhibitors apicularen and archazolid [18]. Due to their known function, compounds spirangien B and soraphen F were the most interesting hits from Part 1 of the screen. Spirangien B is an inhibitor of IkBα, a key regulator of the NF-κB signaling pathway [20]. Repressing IkBα has been also shown to inhibit HIV in Jurkat cells [21]. Soraphens are acetyl-CoA carboxylate inhibitors [22]. This enzyme is also suggested to play a role in HIV infection [23] and other viruses requiring fatty acids for replication [24].

**Table 1:** Robust Z-score ranking of the myxobacterial hits for both part 1 (upper) and part 2 (bottom) of the screen

**Hits from part 1**			**Infectivity**			**Viability**	
**Name**	**Abbr.**	**Z-score**	**% Mean**	**SD**	**Z-score**	**% Mean**	**SD**
Tubulysin l*	Tlsl	-2.47	3.70	1.27	-0.08	92.04	4.38
Kulkenon	Kulk	-2.41	5.15	1.09	0.03	93.01	18.62
Epothilon A*	EpoA	-2.38	0.49	0.15	0.24	87.72	10.27
Spirangien B	SpiB	-2.38	1.09	0.19	0.08	85.71	4.14
Epothilon C*	EpoC	-2.37	0.54	0.14	-0.25	81.63	4.07
Epothilon D*	EpoD	-2.37	1.52	0.54	1.00	97.33	11.03
Terrestribisamid A	TerA	-2.35	6.70	0.26	0.34	95.99	10.58
Enfuvirtide (Control)	T20	-2.30	2.26	0.18	1.36	97.21	0.05
Sulfangolid C	SulfC	-2.29	8.13	1.26	1.73	109.20	13.03
Zidovudine (Control)	AZT	-2.24	4.29	0.21	1.59	98.62	1.59
Apicularen B*	ApiB	-1.93	13.77	2.40	-0.16	82.77	4.37
Disorazol A4*	DszA4	-1.78	20.37	2.01	0.21	94.79	14.99
Tubulysin B*	TlsB	-1.61	24.04	1.01	0.38	89.55	10.43
Disorazol B1*	DszB1	-1.55	25.89	3.60	1.25	104.59	15.96
Disorazol E1*	DszE1	-1.54	26.22	3.20	-0.13	91.48	18.83
Stigmatelin A	StgA	-1.43	29.56	2.80	-0.34	80.43	3.79
Tubulysin A*	TlsA	-1.42	29.95	4.87	-0.11	83.33	9.78
Archazolid A*	ArcA	-1.40	30.47	4.01	-0.35	80.30	4.86
Tubulysin G*	TlsG	-1.34	32.46	3.73	0.34	88.98	2.54
Soraphen F	SorphF	-1.32	31.43	7.72	1.58	107.80	6.05
Disorazol E3*	DszE3	-1.31	31.74	4.50	-0.05	92.32	3.12
Maltepolid B	MalB	-1.16	35.34	8.71	-0.11	91.76	7.42
Crocacin A	CroA	-1.06	41.32	6.39	0.15	86.66	2.43
Noricumazol B	NoricB	-1.01	43.03	6.34	0.86	95.47	5.54
Socein A	SoceinA	-0.97	40.09	7.31	0.68	99.20	13.69
**Hits from part 2**			**Infectivity**			**Viability**	
**Name**	**Abbr.**	**Z-score**	**% Mean**	**SD**	**Z-score**	**% Mean**	**SD**
Rhizopodin A	RhiA	-2.11	0.00	0.00	-1.38	67.39	11.37
Indinavir (control)	IND	-1.93	2.73	0.10	1.43	91.21	8.50
Leupyrrin B1	LeuB1	-1.85	3.12	0.24	-0.01	67.54	11.23
Crocapeptin B*	CrpB	-1.80	11.38	1.78	-1.40	67.17	6.95
Soraphen A	SorA	-1.49	22.24	2.69	0.15	86.61	6.68
Cruentaren A	CruA	-1.25	31.05	5.38	0.66	93.01	5.04

Corresponding % relative infectivity and cell viability is shown. Compounds are listed by infectivity Z-scores. Control drugs are shown for comparison. *: Compounds with anti-HCV activities according to [40].

**Figure 4:**
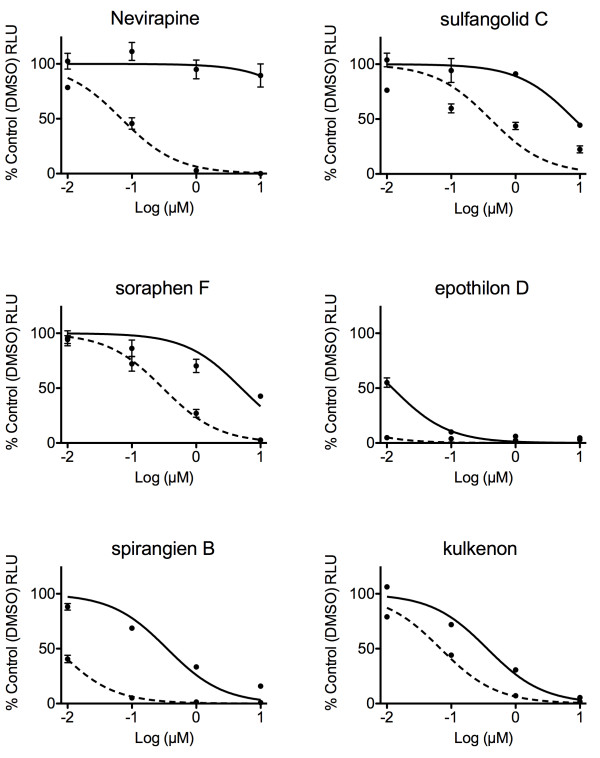
**Dose response curves of five of the HIV hits.** TZM-bl cells were pre-incubated with 10-fold serial dilutions of compounds and vehicle control for 2 h and infected with HIV_LAI_ at an MOI = 0.5. 48 h after initial infection luciferase expression was quantified (see Materials and Methods). Cell toxicity was measured in parallel plates. Curves show the drug dose vs. the response normalized to the control. Error bars are standard error of the mean.

In order to further analyse the results of the primary screen, we performed dose-response assays in TZM-bl cells with five of the initial hits and calculated their Selectivity Index (SI), i.e. antiviral potency, based on their respective effective concentration 50 (EC_50_) and cytotoxic concentration 50 (CC_50_). As shown in Figure 4 and Table 2 the non-nucleoside reverse transcriptase inhibitor Nevirapine, used as control, produced anti-HIV SI values similar to those previously described [25]. The myxobacterial compounds sulfangolid C and soraphen F showed EC_50_ and CC_50_ values comparable to each other, with SI ranging from 16 to 20. Both epothilon D and spirangien B showed lower EC_50_ values than Nevirapine, while spirangien exhibited the highest SI value of the five tested myxobacteria metabolites (>50) (Figure 4 and Table 2). Kulkenon was not selective in the follow-up, with a low SI of around 5.

**Table 2:** Compound names, structures, EC_50_, CC_50_ and SI values for five of the preliminary hits

**Compound name**	**Structure**	**EC**_**50 **_**(μM)**	**CC**_**50 **_**(μM)**	SI*
Nevirapine		0.07	81.8	>1000
Sulfangolid C		0.41	8.18	20.2
Soraphen F		0.30	5.02	16.5
Epothilon D		0.0005	0.012	24.4
Spirangien B		0.007	0.35	52
Kulkenon		0.07	0.36	5.3

*SI: Selectivity Index.

The most interesting hit from Part 2 of the screen was rhizopodin, an actin inhibitor isolated from the myxobacterium *Myxococcus stipitatus*[26]. Actin filaments are known to be essential for virological synapse formation and HIV cell-to-cell transmission, the main route of HIV infection in naïve cells *in vivo*[27,28]. Another hit was leupyrrin B1, a macrodiol with a unique pyrrole ring that has been shown to inhibit DNA, RNA and protein synthesis in yeast [29] (Figure 3 and Table 1). Interestingly, highly substituted pyrroles have been shown to target the HIV reverse transcriptase [30] and, in our primary screen, leupyrrin B1 also showed a mild effect on Part 1 (data not shown). However, the studies needed to test the real potency of these late-acting hits are beyond this report.

In summary, we have established a two-step HTS platform based on the TZM-bl cell line for the screening of novel HIV inhibitors. The advantage of this method is that it can distinguish between compounds acting on early or late events of the HIV replication cycle so that initial hints on the mode-of-action can be already obtained from primary screens. With this assay, a small library of secondary metabolites derived from myxobacteria was screened and interesting primary hits were found. In general, the intrinsic antiviral potency was lower in the follow-up experiments. Unfortunately, these observations are common in HTS campaings, from which few initial hits are confirmed in subsequent assays [31,32]. In particular, comparative low SI values have been commonly observed for natural products of diverse origin [33-38], and it has also been noted that a compound´s biological efficacy is not due to in vitro toxicity when the SI > 10 [39]. In our experiments, even when dose response assays with five of the initial hits showed a rather low SI compared to a standard FDA-approved drug, four compounds exhibited SI values >15. The screening for natural compounds allows for the discovery of molecules with good effectiveness, which could be further optimized by synthetic methods. The myxobacteria secondary metabolism comprises compounds with large chemical components that might "mask" the actual antiviral pharmacophore (see structures in Table 2). Therefore, further structural studies of our initial hits might help recognize smaller drug-like derivatives that in turn could be synthesized and further optimized.

## Conclusions

We have established a robust high-throughput anti-HIV assay that allows, already from the primary screen, to discriminate compounds acting on early and/or late steps of the virus replication cycle. We identified several secondary metabolites derived from myxobacteria with good anti-HIV profiles. Remarkably, compared to a previous screen for Hepatitis C Virus (HCV) using the same library, we found a high amount of overlapping hits (Table 1 and [40]), suggesting that these compounds may target commonly used host factors or pathways necessary for viral replication. Although the intrinsic antiviral potency of most of these compounds remains to be elucidated, the unique structures and mode-of-actions of these natural compounds make myxobacteria an attractive source of chemicals for the development of broad-spectrum antivirals [14].

## Materials and methods

### Cells and culture medium

TZM-bl, a modified HeLa cell-line susceptible to infection with different HIV-1 isolates, was obtained from the NIH AIDS Research and Reference Reagent Program, Cat# 8129) and maintained in Dulbecco’s modified Eagle’s medium (DMEM; Invitrogen, Karlsruhe, Germany) supplemented with 10% heat-inactivated FCS, HEPES 25 mM and 0.5% gentamycin. PM1 cells (NIH AIDS Research and Reference Reagent Program, Cat# 3038) were maintained with RPMI medium supplemented with 10% heat-inactivated FCS and 1% of penicillin-streptomycin. Both cell-lines were cultured at 37°C, 5% CO_2_.

### Virus stocks and infections

HIV-1_LAI_ isolate was obtained from the Centre for AIDS Reagents, NIBSC, UK. Virus was propagated in PM1 cells and titrated in TZM-bl cells as described [41]. 1 mL aliquots of virus stocks were stored at -80°C until use. Infection experiments in TZM-bl cells were performed in quadruple at a multiplicity of infection (MOI) of 0.5. For drug-response assays TZM-bl cells were plated (10^4^ cells/well) in Nunc® MicroWell 96 well optical bottom plates (Sigma) and incubated for 1 h with increasing concentrations of test compounds in 10-fold dilutions or with the corresponding vehicle (DMSO or MeOH) as negative control in triplicates. After drug incubation, cells were infected with HIV_LAI_ and 48 h after infection luciferase activity was measured using Britelite Plus™ (PerkinElmer, Waltham, USA). In parallel, cell viability of TZM-bl cells was determined with an ATP quantification method using the commercial kit CellTiter-Glo® Luminescent Cell Viability Assay (Promega, Madison, USA). ATP is a marker of the presence of metabolically active cells [42]. Therefore, the ATP levels relative to the untreated control are a measure of drug-induced cytotoxicity. Mean luciferase values were normalized to untreated controls and Effective Concentration 50 (EC_50_) and Cytotoxic Concentration 50 (CC_50_) were calculated in GraphPad Prism (GraphPad Software, San Diego, CA, USA) by analyzing the log_dose_ vs. normalized response. The Selectivity Index (SI) refers to the antiviral potency of a drug and is calculated as the ratio of CC_50_ to EC_50_[43,44].

### Test compounds

The library of 154 myxobacterial secondary metabolites used for the screening belongs to a collection of natural compounds isolated at the Helmholtz Centre for Infection Research, Braunschweig, Germany [11,12,45]. Compounds of > 95% purity as measured by LC-MS were provided in 96-well screening plates in a concentration of 1 mM in puriss.p.a. dimethyl sulfoxide (DMSO) or methanol (MeOH). The fusion inhibitor Enfuvirtide (Fuzeon, Roche, Basel Switzerland) and the nucleoside reverse transcriptase inhibitor Zidovudine (NIH AIDS Research and Reference Reagent Program, Cat# 3485) were used as positive controls at final concentrations of 1 μM.

### Two-step TZM-bl based high-throughput screening assay

For the Part 1 of the screen, TZM-bl cells were seeded in 384-well plates at a density of 2500 cells per well in 30 μL of culture medium and incubated overnight at 37°C and 5% CO_2_. After incubation, 50 to 70 nL of the test compounds and DMSO and/or MeOH controls from the 96-well screening plates were dispensed in quadruple to the cells with the PinTool of an Evolution P3 pipetting platform (PerkinElmer, Zaventem, Belgium). Final concentration of compounds was around 1.5 to 2 μM with a content of around 0.2% of DMSO or MeOH in all cases. DMSO or MeOH alone were used as controls. 2 h after addition of compounds, cells were infected with 50 μL of HIV_LAI_ at a MOI of 0.5. In parallel, duplicate plates were left uninfected for quantification of compound-related toxicity. Plates were incubated at 37°C and 5% CO_2_. Forty-eight hours after virus addition, 50 μL supernatant from cells of Part 1 was transferred to fresh TZM-bl cells seeded in 384-wells the day before. Cells of Part 1 plates were assayed for Tat-dependent luciferase expression by adding an equal volume of Britelite Plus™ (PerkinElmer, Waltham, USA) according to the manufacturer´s instructions. Compound-related toxicity in duplicate plates was quantified with the CellTiter-Glo® Luminescent Cell Viability Assay (Promega, Madison, USA) according to the manufacturer´s instructions. ATP is a marker of the presence of metabolically active cells [42]. Following the same procedure, cells from Part 2 of the screen were assayed forty-eight hours after supernatant addition. Luciferase expression was measured with a TECAN Infinite M1000PRO microplate reader (Tecan, Switzerland). The possible outcomes of the screen are depicted in Figure S1.

### Data analysis and statistics

Data was analyzed by obtaining the % mean luciferase values normalized to solvent controls (% control mean RLU) and by calculating the assay Z-factor (a measure for assay quality) and samples (robust) Z-scores (a measure of hit quality) as described [9,10,16]. Briefly, background levels were substracted from the mean luciferase of samples and values were normalized to those of the solvent controls (set to 100% expression). The assay Z-factor was calculated by dividing 3X standard deviations of controls by the sum of their means as described (see Table 1 in [10]). For hit determination, we adapted a previously described robust Z-score calculation [9] to adjust for interplate variation by dividing the absolute deviation of the mean of the quadruple data points (4 wells for each compound) by the median absolute deviation of each plate. Unless stated otherwise, errors are given as ± SD.

## Competing interest

The authors declare that they have no competing interests

## Authors’ contributions

JPM and BH established and performed the screening assay; JPM and EFS performed drug-response assays; HS and RJ provided the myxobacterial library and additional compounds; JPM, JD, RF, FS and AM designed the experimental approach and wrote the manuscript. All listed authors read and approved the final manuscript.

## References

[B1] Arts EJ Hazuda DJ HIV-1 antiretroviral drug therapy Cold Spring Harbor perspectives in medicine 2012 2 4 a007161 10.1101/cshperspect.a007161 22474613 PMC3312400

[B2] Back D New drug interactions in HIV and HCV Retrovirology 2012 9 I8 10.1186/1742-4690-9-S1-I8

[B3] de Maat MM Ekhart GC Huitema AD Koks CH Mulder JW Beijnen JH Drug interactions between antiretroviral drugs and comedicated agents Clinical pharmacokinetics 2003 42 223 282 10.2165/00003088-200342030-00002 12603174

[B4] McIlleron H Meintjes G Burman WJ Maartens G Complications of antiretroviral therapy in patients with tuberculosis: drug interactions, toxicity, and immune reconstitution inflammatory syndrome J Infect Dis 2007 196 S63 S75 10.1086/518655 17624828

[B5] Tseng A Foisy M Important drug-drug interactions in HIV-infected persons on antiretroviral therapy: an update on new interactions between HIV and non-HIV drugs Current infectious disease reports 2012 14 67 82 10.1007/s11908-011-0229-1 22125049

[B6] Wei X Decker JM Liu H Zhang Z Arani RB Kilby JM Saag MS Wu X Shaw GM Kappes JC Emergence of resistant human immunodeficiency virus type 1 in patients receiving fusion inhibitor (T-20) monotherapy Antimicrob Agents Chemother 2002 46 1896 1905 10.1128/AAC.46.6.1896-1905.2002 12019106 PMC127242

[B7] Montefiori DC Evaluating neutralizing antibodies against HIV, SIV, and SHIV in luciferase reporter gene assays Curr Protoc Immunol 2005 Chapter 12 Unit 12 11 10.1002/0471142735.im1211s64 18432938

[B8] Brass AL Dykxhoorn DM Benita Y Yan N Engelman A Xavier RJ Lieberman J Elledge SJ Identification of host proteins required for HIV infection through a functional genomic screen Science 2008 319 921 926 10.1126/science.1152725 18187620

[B9] Tan X Hu L Luquette LJ 3rd Gao G Liu Y Qu H Xi R Lu ZJ Park PJ Elledge SJ Systematic identification of synergistic drug pairs targeting HIV Nat Biotechnol 2012 30 1125 1130 10.1038/nbt.2391 23064238 PMC3494743

[B10] Zhang JH Chung TD Oldenburg KR A simple statistical parameter for use in evaluation and validation of high throughput screening assays J Biomol Screen 1999 4 67 73 10.1177/108705719900400206 10838414

[B11] Reichenbach H Myxobacteria, producers of novel bioactive substances J Ind Microbiol Biotechnol 2001 27 149 156 10.1038/sj.jim.7000025 11780785

[B12] Reichenbach H Höfle G Myxobacteria as producers of secondary metabolites Drug discovery from nature 1999 149 179

[B13] Pandey S Sree A Dash SS Sethi DP Chowdhury L Diversity of marine bacteria producing beta-glucosidase inhibitors Microb Cell Fact 2013 12 35 10.1186/1475-2859-12-35 23590573 PMC3639877

[B14] Diez J Martinez JP Mestres J Sasse F Frank R Meyerhans A Myxobacteria: natural pharmaceutical factories Microb Cell Fact 2012 11 52 10.1186/1475-2859-11-52 22545867 PMC3420326

[B15] Huttel S Muller R Methods to optimize myxobacterial fermentations using off-gas analysis Microb Cell Fact 2012 11 59 10.1186/1475-2859-11-59 22571441 PMC3445963

[B16] Malo N Hanley JA Cerquozzi S Pelletier J Nadon R Statistical practice in high-throughput screening data analysis Nat Biotechnol 2006 24 167 175 10.1038/nbt1186 16465162

[B17] Hopkins CD Wipf P Isolation, biology and chemistry of the disorazoles: new anti-cancer macrodiolides Nat Prod Rep 2009 26 585 601 10.1039/b813799b 19387496 PMC2711774

[B18] Weissman KJ Muller R Myxobacterial secondary metabolites: bioactivities and modes-of-action Nat Prod Rep 2010 27 1276 1295 10.1039/c001260m 20520915

[B19] Reichenbach H Hofle G Discovery and development of the epothilones: a novel class of antineoplastic drugs Drugs R D 2008 9 1 10 18095749 10.2165/00126839-200809010-00001 PMC7044396

[B20] Reboll MR Ritter B Sasse F Niggemann J Frank R Nourbakhsh M The myxobacterial compounds spirangien a and spirangien M522 are potent inhibitors of IL‒8 expression ChemBioChem 2012 13 409 415 10.1002/cbic.201100635 22271561

[B21] Kwon H Pelletier N DeLuca C Genin P Cisternas S Lin R Wainberg MA Hiscott J Inducible expression of IkappaBalpha repressor mutants interferes with NF-kappaB activity and HIV-1 replication in Jurkat T cells J Biol Chem 1998 273 7431 7440 10.1074/jbc.273.13.7431 9516441

[B22] Jump DB Torres-Gonzalez M Olson LK Soraphen A, an inhibitor of acetyl CoA carboxylase activity, interferes with fatty acid elongation Biochemical pharmacology 2011 81 649 660 10.1016/j.bcp.2010.12.014 21184748 PMC3031740

[B23] Steger DJ Eberharter A John S Grant PA Workman JL Purified histone acetyltransferase complexes stimulate HIV-1 transcription from preassembled nucleosomal arrays Proc Natl Acad Sci 1998 95 12924 12929 10.1073/pnas.95.22.12924 9789016 PMC23656

[B24] Heaton NS Perera R Berger KL Khadka S LaCount DJ Kuhn RJ Randall G Dengue virus nonstructural protein 3 redistributes fatty acid synthase to sites of viral replication and increases cellular fatty acid synthesis Proc Natl Acad Sci 2010 107 17345 17350 10.1073/pnas.1010811107 20855599 PMC2951450

[B25] Koh Y Haim H Engelman A Identification and characterization of persistent intracellular human immunodeficiency virus type 1 integrase strand transfer inhibitor activity Antimicrobial agents and chemotherapy 2011 55 42 49 10.1128/AAC.01064-10 21060108 PMC3019619

[B26] Sasse F Steinmetz H Höfle G Reichenbach H Rhizopodin, a new compound from myxococcus stipitatus (myxobacteria) causes formation of rhizopodia-like structures in animal cell cultures. Production, isolation, physico-chemical and biological properties J Antibiot (Tokyo) 1993 46 741 10.7164/antibiotics.46.741 8514628

[B27] Felts RL Narayan K Estes JD Shi D Trubey CM Fu J Hartnell LM Ruthel GT Schneider DK Nagashima K 3D visualization of HIV transfer at the virological synapse between dendritic cells and T cells Proc Natl Acad Sci 2010 107 13336 13341 10.1073/pnas.1003040107 20624966 PMC2922156

[B28] Jolly C Kashefi K Hollinshead M Sattentau QJ HIV-1 cell to cell transfer across an Env-induced, actin-dependent synapse J Exp Med 2004 199 283 293 10.1084/jem.20030648 14734528 PMC2211771

[B29] Bode HB Irschik H Wenzel SC Reichenbach H Muller R Hofle G The leupyrrins: a structurally unique family of secondary metabolites from the myxobacterium Sorangium cellulosum J Nat Prod 2003 66 1203 1206 10.1021/np030109v 14510597

[B30] Antonucci T Warmus J Hodges J Nickell D Characterization of the antiviral activity of highly substituted pyrroles: a novel class of non-nucleoside HIV-1 reverse transcriptase inhibitor Antiviral chemistry & chemotherapy 1995 6 98 10824077100

[B31] Malo N Hanley JA Cerquozzi S Pelletier J Nadon R Statistical practice in high-throughput screening data analysis Nat Biotechnol 2006 24 2 167 175 10.1038/nbt1186 16465162

[B32] Smith T Ho P-i Yue K Itkin Z MacDougall D Paolucci M Hill A Auld DS Comparison of compound administration methods in biochemical assays: effects on apparent compound potency using either assay-ready compound plates or pin tool -delivered compounds J Biomol Screen 2013 18 1 14 25 10.1177/1087057112455434 22904199

[B33] Asres K Bucar F Kartnig T Witvrouw M Pannecouque C De Clercq E Antiviral activity against human immunodeficiency virus type 1 (HIV‒1) and type 2 (HIV‒2) of ethnobotanically selected Ethiopian medicinal plants Phytother Res 2001 15 62 69 10.1002/1099-1573(200102)15:1<62::AID-PTR956>3.0.CO;2-X 11180526

[B34] Cos P Vlietinck AJ Berghe DV Maes L Anti-infective potential of natural products: how to develop a stronger in vitro 'proof-of-concept’ Journal of ethnopharmacology 2006 106 290 302 10.1016/j.jep.2006.04.003 16698208

[B35] Crance JM Scaramozzino N Jouan A Garin D Interferon, ribavirin, 6-azauridine and glycyrrhizin: antiviral compounds active against pathogenic flaviviruses Antiviral research 2003 58 73 79 10.1016/S0166-3542(02)00185-7 12719009

[B36] Fiore C Eisenhut M Krausse R Ragazzi E Pellati D Armanini D Bielenberg J Antiviral effects of Glycyrrhiza species Phytother Res 2008 22 141 148 10.1002/ptr.2295 17886224 PMC7167979

[B37] Hayashi K Minoda K Nagaoka Y Hayashi T Uesato S Antiviral activity of berberine and related compounds against human cytomegalovirus Bioorganic & medicinal chemistry letters 2007 17 1562 1564 10.1016/j.bmcl.2006.12.085 17239594

[B38] Sun Y Song M Niu L Bai X Sun N Zhao X Jiang J He J Li H Antiviral effects of the constituents derived from Chinese herb medicines on infectious bursal disease virus Pharmaceutical biology 2013 51 9 1127 1143 10.3109/13880209.2013.781197 23607905

[B39] Vonthron-Sénécheau C Weniger B Ouattara M Bi FT Kamenan A Lobstein A Brun R Anton R In vitro antiplasmodial activity and cytotoxicity of ethnobotanically selected Ivorian plants Journal of ethnopharmacology 2003 87 221 225 10.1016/S0378-8741(03)00144-2 12860312

[B40] Gentzsch J Hinkelmann B Kaderali L Irschik H Jansen R Sasse F Frank R Pietschmann T Hepatitis C virus complete life cycle screen for identification of small molecules with pro- or antiviral activity Antiviral Res 2011 89 136 148 10.1016/j.antiviral.2010.12.005 21167208

[B41] Montefiori DC Evaluating neutralizing antibodies against HIV, SIV, and SHIV in luciferase reporter gene assays Current protocols in immunology 2005 Chap.12 Unit 12.11 18432938 10.1002/0471142735.im1211s64

[B42] Crouch SP Kozlowski R Slater KJ Fletcher J The use of ATP bioluminescence as a measure of cell proliferation and cytotoxicity J Immunol Methods 1993 160 81 88 10.1016/0022-1759(93)90011-U 7680699

[B43] Tamamura H Omagari A Oishi S Kanamoto T Yamamoto N Peiper SC Nakashima H Fujii N Pharmacophore identification of a specific CXCR4 inhibitor, T140, leads to development of efective anti-HIV agents with very high selectivity indexes Bioorg Med Chem 2000 10 2633 2637 10.1016/S0960-894X(00)00535-7 11128640

[B44] Pauwels R Andries K Debyser Z Van Daele P Schols D Stoffels P De Vreese K Woestenborghs R Vandamme aM Janssen CG Potent and highly selective human immunodeficiency virus type 1 (HIV-1) inhibition by a series of alpha-anilinophenylacetamide derivatives targeted at HIV-1 reverse transcriptase PNAS 1993 90 1711 1715 10.1073/pnas.90.5.1711 7680476 PMC45949

[B45] Reichenbach H Myxobacteria Encyclopedia of Bioprocess Technology 1992 Vol. 1-5 Wiley-Interscience

